# Avoiding Misdiagnosis in Patients with Neurological Emergencies

**DOI:** 10.1155/2012/949275

**Published:** 2012-07-25

**Authors:** Jennifer V. Pope, Jonathan A. Edlow

**Affiliations:** Department of Emergency Medicine, Harvard Medical School, Beth Israel Deaconess Medical Center, Boston, MA 02215, USA

## Abstract

Approximately 5% of patients presenting to emergency departments have neurological symptoms. The most common symptoms or diagnoses include headache, dizziness, back pain, weakness, and seizure disorder. Little is known about the actual misdiagnosis of these patients, which can have disastrous consequences for both the patients and the physicians. This paper reviews the existing literature about the misdiagnosis of neurological emergencies and analyzes the reason behind the misdiagnosis by specific presenting complaint. Our goal is to help emergency physicians and other providers reduce diagnostic error, understand how these errors are made, and improve patient care.

## 1. Introduction

Approximately 5% of emergency department (ED) patients present with neurological symptoms [1]. The most common symptoms or diagnoses that these patients have are headache, dizziness, back pain, weakness, and seizure disorders [2–6]. In recent years, improved time-dependent treatments for patients with acute neurological emergencies have been developed, increasing the importance of a rapid and accurate diagnosis. Underdiagnosis may have disastrous consequences. Conversely, overtesting leads to inefficient resource utilization that is undesirable for both economic and medical reasons.

## 2. Methods

A PubMed search on February 8, 2012 for the intersection of “misdiagnosis” and “neurological emergency” as title or abstract words resulted in 88 results. In addition to this literature review, we will incorporate experience from over 30 years of ED clinical practice, teaching medical students, and residents, over a decade of evaluation of medicolegal cases, and analyzing diagnostic errors committed by our colleagues and ourselves in peer review.

We review the existing literature about misdiagnosis of nontraumatic neurological emergencies in general, and then by specific presenting complaints. We conclude by analyzing the reasons for misdiagnosis. Our goals are to help emergency physicians (EPs) and other front-line clinicians reduce misdiagnosis of patients with neurological emergencies and to be hypothesis generating so that we can better study and understand misdiagnosis in these patients and to improve patients' clinical outcomes.

## 3. Results

### 3.1. General Studies about Misdiagnosis of Neurological Emergencies

Few high-quality data on the subject of ED misdiagnosis of patients with neurological emergencies exist. Most papers on misdiagnosis of patients with neurological emergencies focus on patients with a particular diagnosis or presenting symptom. Only a few analyze the general topic of all-comers with neurological symptoms [2–4]. There are methodological problems with all of these articles. The EP's diagnosis is made earlier in a patient's course. Therefore, less historical information is usually available, the natural course of the disease process is less well defined, and almost always, fewer results of diagnostic testing are available. The primary job of the EP is to ensure clinical stability and proper disposition of a patient, both of which are possible without necessarily making a specific etiologic diagnosis. Therefore, EP's charted diagnosis is often a tentative one, or even simply a repetition of the major symptom or sign. Neurologists on the other hand appropriately try to make a specific diagnosis.

For all these reasons, the comparisons being made are not equivalent. In addition, one must account for the underlying infrastructure of emergency services where the study was done. Some data originates from Europe where patients with acute neurological emergencies are often triaged directly to neurologists or the “EP” is actually a prehospital provider. The training of an EP differs across these locales.

Another limitation of all the studies is that they only examine those patients whom the EP decided to consult the neurologist; many patients with clear-cut diagnoses (e.g., peripheral 7th nerve palsy or benign paroxysmal positional vertigo (BPPV)) may have been well managed without neurological consultation. Thus, the frequency of misdiagnosis of patients who did not have a neurology consultation is unknown. The ideal study would compare diagnostic accuracy of similar patients at the same phase of their care and using the same diagnostic information. Of course it is very unlikely that such a study will ever be done.

A frequently cited article by Moulin and colleagues tried to assess the impact of neurology consultants on the outcome of 1679 patients with neurological emergencies in a large French ED [4]. Neurology consults were obtained in 14.7% of all patients. They found that there was a complete change in diagnosis in 52.5% of cases. They included both false positive (e.g., the EP diagnosed stroke, but the patient had a tumor) and false negative (e.g., EP diagnosed benign vertigo but the patient had a stroke) diagnoses. By design, the EPs were blinded to the study that the neurologists had planned and executed, clearly introducing potential bias. More importantly, the neurologists' diagnoses were made after access to neurological tests such as computed tomography (CT), magnetic resonance imaging (MRI), lumbar puncture (LP), electroencephalogram (EEG), and others, which “could not have been previously conducted by the ER team.” It is hardly surprising that many diagnoses would change based on adding all of those diagnostic modalities to the history and physical examination. Finally, the training of these emergency physicians is not specified. These methodological flaws make this article irrelevant to modern EM practice, at least in North America.

In a large Canadian ED, Moeller and colleagues studied 493 patients with neurological emergencies who had a neurologist consult in the ED [3]. In 60.4% of cases, the ED diagnosis was the same as the final diagnosis. In 19.1% of cases, there was frank disagreement and in another 16.6%, there was “significant uncertainly” between the two diagnoses. Importantly, the “gold standard” diagnosis for patients who were admitted or had neurologic followup was the final hospital discharge diagnosis and the ultimate outpatient neurological diagnosis respectively. When they compared the consulting ED neurologist's diagnosis with the final diagnosis, there was agreement in 80% of cases. Some of the patients were referred by family practitioners or other hospitals. The investigators found that diagnoses made by EPs were more likely to be concordant with the final diagnoses than compared with the ones made by the other sources. The vast majority of the diagnostic error was over-diagnosis.

Two other studies did not so much compare EP versus neurologists' diagnoses, as categorize the types of ED neurological emergencies [2, 5]. In all studies of emergency neurological consultations, stroke, headache disorders, seizures, and dizziness make up a large majority of the patients [2–5, 7]. In Hansen's study, which analyzed 500 neurology consultations at a tertiary U.S. academic hospital, 4.8% of total ED patients had a neurology consultation (1/3 of the number in the French study). The mean length of stay for those patients was 7.4 hours (significantly longer than for the average ED patient—4.9 hours) and remarkably similar to the “just under 8 hours” in the Canadian study. In the latter study, it is interesting to note that patients with diagnostic ambiguity stayed in the ED much longer than those where there was either agreement or disagreement (between consulter and consultant) about the diagnosis.

Although all these studies have limitations, there is a common theme that runs through them. Diagnosis of patients with neurological emergencies is imperfect. There is significant underdiagnosis (which threatens patient safety) and overtesting (which wastes resources). Patients with stroke, dizziness, headache, and seizures are the most common sources for these errors. In a study of unplanned ED return visits, many of which were due to missed diagnoses, headache and vertigo were among the most common presenting symptoms [8]. Apart from the studies discussed above, most others have analyzed misdiagnosis by either specific presenting symptoms (e.g., headache) or by specific diagnosis (e.g., SAH).

### 3.2. Headache

Headache accounts for roughly 2% of ED visits, of which only a very small percentage have serious secondary causes [9]. This “needle in the haystack” phenomenon may lead clinicians to not consider serious secondary causes. Deciding which patients to investigate beyond clinical evaluation can be difficult; history and physical examination must focus on uncovering “red flags” that suggest the need for further testing [10].

Much of the literature about misdiagnosis of headache focuses on subarachnoid hemorrhage (SAH) [11–13]. While older literature showed a misdiagnosis rate from 12–25%, the latest data based on misdiagnosis restricted to the ED puts that figure at 5% [13]. Recurring reasons for misdiagnosis include not considering the full spectrum of presentations, not following an algorithmic workup and not understanding the limitations of the tests in that workup [11, 12].

Regarding presentation, not all patients with SAH have a truly abrupt onset of their headache [14]. In some patients, the headache improves after analgesics including triptans [15]. Some patients present with prominent vomiting or fever and neck pain or with hypertension, each of which can divert the physician's diagnosis to other less serious problems such as gastroenteritis, viral syndrome, or hypertensive crisis [11, 12]. Patients with SAH do not necessarily “look ill,” have any neurological deficits or meningism.

Even in the less acuity-skewed population of a neurology practice, some argue for a lower threshold for imaging in patients with new-onset headache [16]. This is probably even more important in an ED practice, where the incidence of secondary causes may be higher. In ED populations, patients with thunderclap headache have an incidence of SAH of between 8–16% [11, 14, 17]. In one large series of misdiagnosed SAH, failure to do a CT scan was the most common error [18]. However, a negative CT may not exclude SAH, especially if performed after 6 hours from headache onset, and if a CT shows findings of chronic sinusitis, physicians may inappropriately stop the work-up and diagnose sinusitis as a cause of an acute headache, which is actually very uncommon [17, 19, 20].

It is not surprising that patients with less common causes of headache may be initially misdiagnosed. Most patients with headache due to a brain tumor have no distinguishing pain characteristics [21, 22], although persistent vomiting with headache, especially if associated with lethargy, suggests obstructive hydrocephalus [23]. Patients with other uncommon causes of headache, such as cerebral venous sinus thrombosis (CVST) and cervicocranial arterial dissections are frequently misdiagnosed on the first physician encounter. These problems may also present with isolated headache without specific qualities in patients without risk factors [24, 25]. For these diagnoses and other uncommon ones, the issue of diagnosing a rare condition without major distinguishing features presents obvious difficulties.

### 3.3. Dizziness

As with headache, dizziness has both benign and serious causes that can be difficult to distinguish from one another. Diagnosis of the dizzy patient is inherently fraught with problems. The diagnostic algorithms that doctors are taught may be flawed. Increasing evidence suggests that the traditional “symptom quality” approach (“what do you mean by “dizzy”?”) is less effective that a new “timing and triggers” approach, in which the physician asks about the temporal characteristics of the symptoms [26–28]. Data suggests that whether the patient uses the word “vertigo” or “spinning” versus “dizzy” or “lightheaded” is not so useful in determining etiology. “Vertigo” versus nonspecific dizziness does not help predict etiology in dizzy patients [29, 30]. For example, patients with BPPV often use nonspecific (non-vertigo) descriptors for their symptoms [31] and patients with clear-cut cardiac causes of dizziness often complain of “vertigo” [32].

Because of the prevailing paradigm, EPs may have an overly generalized approach to dizzy patients [28]. There is also a significant overlap between the presentations of benign (vestibular neuritis and labyrinthitis) from serious (cerebellar and brainstem stroke) presentations [33, 34]. Deficits in physician knowledge may also contribute, for example, documentation of nystagmus is often inaccurate [35]. Finally, lack of understanding of the limitations of neuroimaging is another issue. Some EPs incorrectly believe in the sensitivity of CT to exclude posterior circulation stroke [28], which may also be undetected by MRI in the first 48 hours [33].

Neurologists may also have difficulty diagnosing dizzy patients in the ED. Royl reported on 475 patients seen in a German neurology ED staffed by neurologists. Of the 124 patients for whom followup was available, 43% of ED diagnoses were “corrected” [36]. Six percent of the patients diagnosed with benign conditions were changed to serious ones and 23% of the serious ones were reversed to benign. In a California study of ED patients discharged with an ICD-9 code compatible with dizziness, there was an increase in the incidence of adverse cerebrovascular events in the next 30 days, suggesting that an important diagnosis had been missed [37].

The most feared misdiagnosis of dizzy patients is stroke. These are usually ischemic strokes of the brainstem and cerebellum. In one series of 240 consecutive cerebellar strokes, 10% presented as an acute vestibular syndrome (AVS) suggesting a peripheral cause [34]. Nearly all of these patients had posterior inferior cerebellar artery strokes. Patients with misdiagnosis may have poor outcomes due to posterior fossa edema and brainstem compression [38]. Distinguishing stroke from benign peripheral causes is critical, not just to treat the acute complications, but also to evaluate and treat the underlying vascular lesion in order to prevent a second event [39].

### 3.4. Back Pain

Along with headache and dizziness, back pain is very common and most patients have benign, self-limited causes. With back pain, there are fewer “needles” in a larger “haystack.” Common causes of cord or cauda equina compression include herniated disk, tumor, abscess, and hematoma. In primary care practices, all four of these etiologies amount to roughly 1% of patients with back pain [40]. Diagnoses generally require MRI, thus setting up the classic tension between resource utilization versus patient outcomes [40]. Surprisingly, few data exist about prevalence in the ED, although it is likely higher due to skewed acuity. Red flags include new pain in patients >age 50 years, a history of cancer, fever, weight loss, an immunocompromised state, intravenous drug use, recent bacteremia or urinary tract infection, pain that is worse with rest or at night, sphincter symptoms, bilateral sciatica, failure to improve over weeks, anticoagulation, and recent spinal procedure [41]. Some patients have no identifiable red flags.

Cauda equina syndrome (CES) can be misdiagnosed and/or lead to malpractice claims because of inadequate history, physical examination, or communication between physicians and between physicians and nurses [42]. In one small series of 32 patients with CES, fewer than 20% presented with the classic presentation of bilateral sciatica, leg weakness, saddle anesthesia, and sphincter dysfunction [43]. The most common reason for misdiagnosis in that series was failure to consider the diagnosis. In a retrospective study of 23 patients with suspected CES, the diagnostic accuracy of individual findings of urinary retention, frequency, incontinence, or altered urinary or perineal sensation ranged from 57–65 percent [44]. In another retrospective study of 58 consecutive patients of suspected CES, having 2 of the following 3 findings (bilateral sciatica, subjective urinary retention, or rectal symptoms) increased the likelihood of a positive MRI 48 folds [45].

Another finding that is often ignored is ataxia or new frequent falls. In a study of 63 patients with nontraumatic cord compression or CES, nearly one in four patients had ataxia or gait difficulty with neither sensory nor motor findings [46]. For spinal epidural abscess (SEA), the incidence of the typical triad of back pain, fever, and neurological deficit is low [47–49]. For all these reasons, misdiagnosis or delayed diagnosis is common [47, 50, 51].

Some algorithms include measuring inflammatory markers such as the erythrocyte sedimentation rate (ESR) or C-reactive protein (CRP). Sensitivity for an ESR (>20 mm/hour) in infectious causes of cord or cauda equina compression such as SEA or vertebral osteomyelitis, range from 76–95%; the corresponding figures for CRP are 82–98% [41, 52, 53]. For neoplastic causes, the sensitivity of ESR (>20 mm/hour) is 78% [54]. For the ESR, increasing the threshold value increases specificity at the cost of sensitivity.

MRI with gadolinium is the test of choice for most of these problems [50, 55]. A significant diagnostic issue is that in many practice settings, obtaining an urgent MRI can be difficult or impossible. In the absence of a strict diagnostic algorithm that forces the clinician to get the MRI, even if that requires a transfer, the lack of availability of the diagnostic gold standard may lead to misdiagnosis. Both SEA and epidural tumor often affect multiple areas of the spine; therefore, it is important to decide which part of the spine to image. Some experts recommend that the entire spine should be imaged [55]. Finally, there are issues of resource utilization; in one study of 106 patients undergoing MRI for possible SEA, only 7 were positive [56].

### 3.5. Weakness

The majority of patients presenting to an ED with generalized weakness have a variety of “toxic-metabolic” problems including electrolyte abnormalities and dehydration, medication side effects, and systemic infections. Acute neurological causes of generalized weakness include uncommon diagnoses such as Guillain-Barré syndrome (GBS), transverse myelitis, myasthenia gravis, and periodic paralysis, as well as rarer conditions such as tick paralysis, botulism, and others.

Misdiagnosis of GBS is common [57, 58]. In a series of 20 ED cases, most patients complained of weakness but some presented with paresthesia [57]. 15 of the 20 patients were incorrectly diagnosed on their first ED visit. Four patients initially presented with sensory symptoms. Six of the 20 had normal cerebrospinal fluid (CSF). The notion that the CSF protein is always elevated is wrong; normal CSF protein is common in the first week of GBS [59]. Misdiagnosis of Lambert-Eaton syndrome is also common [60]. Similarly with myasthenia gravis (MG), the mean time to correct diagnosis is over a year [61]. The variability of specific symptoms and their timing makes diagnosing MG difficult. Therefore, to diagnose MG in the ED, it is essential to know the full spectrum of possible presentations [62]. In transverse myelitis, asymmetric cord involvement can lead to atypical presentations that make diagnosis more difficult [63]. Unless one checks the serum potassium at the time of symptoms, periodic paralysis may be missed. In very rare conditions such as botulism and tick paralysis, initial misdiagnosis is quite common [64, 65].

Serious misdiagnosis can occur in patients with stroke and transient cerebral ischemia (TIA) who present with focal weakness. Various studies have reported that the rate of ED misdiagnosis of stroke varies widely, from as low as 2% to as high as 56% [66–72]. To some extent, the variation relates to study design. The two most recent North American studies found rates of misdiagnosis of roughly 10% [69, 70]. Current diagnostic scoring systems for ischemic stroke emphasize lateralizing motor findings [72–74]. Factors associated with stroke misdiagnosis include young age, posterior circulation or sensory symptoms, and lack of lateralizing weakness [38, 75, 76]. Some stroke patients have NIH stroke scores of zero [77]. Many of the patients in that study had posterior circulation strokes. Finally, physicians must understand that some strokes, even of the anterior circulation, present with atypical symptoms like neuropsychiatric symptoms or abnormal movements at stroke onset [78].

Diagnosis of TIA is more difficult because most TIA patients are neurologically intact by the time they are in the ED. An early study found a misdiagnosis rate by the emergency physician of 6% [79]. A major limitation of this study is that neurologists made their diagnosis by reviewing the ED chart, not by independent clinical evaluation. Another study found a misdiagnosis rate by EPs of 60%, with factors leading to misdiagnosis including gradual onset of symptoms, prior similar episodes, and nonspecific symptoms [80]. The most recent and methodologically sound study found an ED misdiagnosis rate of 36% [81]. It also showed that the presence of headache, involuntary movements, and dizziness were all associated with a non-TIA diagnosis.

Apart from the aforementioned studies of SAH, there has not been much systematic study of misdiagnosis of intracerebral hemorrhage (ICH). Both CT and MRI are very sensitive for ICH; therefore, when physicians perform brain imaging in patients with weakness, they will find ICH when it exists. Although ICH often presents more dramatically than ischemic stroke, there is significant overlap. It is certainly possible that patients who are not imaged will be misdiagnosed.

### 3.6. Seizures

As with TIA, the diagnosis of a seizure often depends entirely upon the history of an event that the physician has not witnessed. Therefore, it is important to try to obtain information from any witnesses of the event, and to gather what data one can from the physical examination to distinguish the causes of these transient episodes of loss of consciousness. The most common issue is distinguishing syncope from seizure, but one must also separate true seizures from pseudoseizures (also referred to as psychogenic seizures and nonepileptic attack disorder). Of these three conditions, syncope is by far the most common.

In one review, the misdiagnosis rate overall for seizures in both children and adults ranged from 5–30% [82]. In adult patients incorrectly diagnosed with seizure, the most common final diagnoses were syncope and pseudoseizures [82, 83]. In children, various benign paroxysmal disorders such as breathing holding spells and night terrors were the most common final diagnoses [82]. The source of misdiagnosis was not entirely from the ED. It is important to note that electroencephalography (EEG) is not specific for seizures and lacks sensitivity. That is to say, an abnormal EEG does not exclude pseudoseizures and a normal EEG does not exclude true seizures or confirm pseudoseizures [84–87].

There are several characteristics that help emergency clinicians to distinguish between syncope, epileptic seizures, and pseudoseizures. Syncope may have a prodromal sensation of warmth, lightheadedness, sweating, and facial pallor, and is often precipitated by various triggers. The event starts rapidly and recovery is prompt. In cardiac causes of syncope, palpitations or chest pain may occur together. Importantly, however, “convulsive syncope,” in which the faint is accompanied by some tonic-clonic jerking due to brain hypoperfusion is common [85, 88, 89]. Tongue biting may occur but it is usually at the tip of the tongue [90]. Urinary incontinence is unusual but may also occur [85, 86].

Patients with true seizures often have a preceding aura or repetitive movements (chewing or lip smacking), lateral biting of the tongue or cheek, facial cyanosis, sphincter incontinence, head turning towards one side, and postictal confusion that is slow to resolve [85, 90, 91]. Postevent neurological examination may show focal deficits [86]. Though not extensively studied, transient anion gap acidosis is also associated with true seizure [92]. Up to 36% of patients with “intractable seizures” actually have pseudoseizures [84]. Patients with pseudoseizures may show side-to-side head movements, changing symptoms if multiple spells, gradual onset and waxing and waning during the spell, rapid recovery, and bizarre movements involving the entire body without any “logical” march [86]. Tongue biting and incontinence are less common in patients with pseudoseizures compared with true seizures [90].

In many ED patients with transient loss of consciousness, a definite diagnosis will not be possible. Coordination of subsequent care for testing not available in an ED such as tilt-table testing, continuous loop ECG monitoring, or video-monitored 48-hour EEG testing may help to reduce misdiagnosis.

### 3.7. Conversion Reaction (Functional Neurological Symptoms)

Conversion reactions overlap the symptom-oriented discussion above. The most common conversion reactions relate to weakness and seizures [93]. One report of ED patients diagnosed with conversion reaction who later proved to have organic disease emphasizes that misdiagnosis often relates to patients having symptoms atypical for organic disease (e.g., “I've never seen anything like this before” or symptoms being “non-anatomic”) [94]. Hoover's sign (weak hip extension that becomes normal on testing contralateral hip flexion) was found to be moderately sensitive and very specific for functional weakness [95]. In the specific setting of possible ischemic stroke, it is obviously preferable to not give thrombolytic therapy to someone who does not have a stroke. However, patients with stroke mimics who are CT negative, have never been reported to have hemorrhagic complications [96–98]. Although a high degree of diagnostic accuracy is possible [99], EPs should be very hesitant to make a diagnosis of conversion reaction in the ED.

## 4. Discussion

Before discussing the data, it is important to acknowledge there limitations. The literature does not contain high-quality data on this subject, and the data derived from our experience with quality assurance and medicolegal case review is by definition skewed towards cases with poor outcomes. We acknowledge that our conclusions are limited by the weakness of the data upon which they are built; however, we believe that this is the best available analysis of the data.

Misdiagnosis contributes to medical malpractice in the ED and patient harm [100, 101]. The underlying reasons included inadequate history and physical examination, failure to order and correctly interpret tests, and failure to obtain a consultation [101]. In Table 1, we have listed potential reasons for misdiagnosis of patients with neurological emergencies. Researchers in the field of diagnostic error often characterize errors in terms of cognitive analysis, which is useful for research [102]. Herein, however, we will categorize reasons for error in everyday terms that average clinicians will not only understand but also relate to.

Time pressures, frequent interruptions, and distractions are common in the ED. For stroke, time pressures related to thrombolytic use force EPs to “diagnose” a stroke within minutes of the patient's presentation, when key historical details may be unavailable. For the less common diagnoses, the “needle in the haystack” phenomenon exists. “Classic” triads and the “typical” symptoms that are emphasized in medical education are often absent. Preconceived notions are sometimes wrong. In addition, examining the nervous system is more complicated than examining the heart or lungs. Charting systems, designed to maximize billing, discourage good documentation. The best test for some conditions, MRI, is often unavailable. Incidental findings on physical exam or imaging tests may distract and prematurely stop the workup. A false normal study (due to interpretation error or imaging the wrong site or at the wrong time) may do the same.

Over testing can also result in patient harm. Incorrect diagnosis of a seizure often leads to anticonvulsant use or driving restrictions. With respect to investigations, ED use of CT more than tripled over the period 1995–2007 [103] and interestingly there is a 3-fold variation of CT use across individual physicians [104]. Apart from obvious issues of diagnostic accuracy, evidence is mounting about the long-term consequences of increasing radiation exposure [105]. Furthermore, incidental findings drive further investigations, which may lead to adverse consequences [106].

Finally, it must be acknowledged that some degree of misdiagnosis is unavoidable [107]. Making every diagnosis every time has costs. Even immediate ED neurological consultation will not lead to diagnostic perfection. Both Moeller and Royl showed that neurologic evaluation in the ED was still associated with some misdiagnosis [3, 36]. In a study of malpractice cases against neurologists, EPs were codefendants in 44% of cases [108]. These data corroborate the obvious conclusion that simply consulting a neurologist does not eliminate potential errors. Because some diagnostic uncertainly is inevitable, explicit communication between physicians, physicians and patients and thoughtful coordination of followup care after the ED phase become critically important.

## 5. Conclusions and Solutions

A full analysis of the reasons behind these potential errors and solutions to the problems is beyond the scope of this review. However, several generic issues exist. Less than 20% of emergency medicine residencies require a neurology rotation [109]. Education is mostly lecture based; however, many of these lessons are best taught by studying real-life patients at the bedside. Important as education is, diagnostic error is frequently not the fault of a misinformed individual. Numerous articles have addressed how to reduce diagnostic errors in medicine, from both practical and research perspectives [110–117]. Some potential components to the solution include better physician education in neurological emergencies that encourage detailed history-taking and systematic physical examination, improved access to supportive diagnostic tests (MRI), real-time neurology consultation and communicate clearly with patients and other physicians who will be seeing them in followup. Further well-designed studies are needed in the area of misdiagnosis of neurological emergencies to improve patient care and the use of healthcare resources.

## Figures and Tables

**Table 1:** Some reasons for misdiagnosis in patients with neurological emergencies.

	Headache	Dizziness	Back Pain	Weakness	Seizure
History	Patients use words like “migraine” and “sinus infection” that may mislead the physician. Beware previous diagnoses; they might be wrong.	The use of the word “vertigo” versus other dizziness descriptors is not etiologically useful.	Patients use word “sciatica” which may lead physicians to diagnose sciatica.	Stroke patients may complain of “clumsiness” or “my arm felt like lead” rather than “weakness”.	Patient (or witness) says “seizure” after a faint Seizure patients often present after the seizure with only an altered mental status or with a postictal “Todd's” paralysis

Physical exam	Patients with SAH may be well appearing and neurologically intact.	Patients with small posterior circulation strokes can mimic a peripheral vestibular presentation.	Patients with serious causes of back pain can present without neurological deficits.	Patients with stroke can present with just about any focal deficit depending upon the occluded vessel. Myasthenia patients' symptoms wax and wane. GBS patients' first symptoms may be purely sensory.	Patients may be lethargic, but neurologically intact.

Diagnostic testing	For SAH, CT sensitivity is good but decays with time. CT has poor sensitivity for CVST and dissection.	CT is a poor test for cerebellar and brainstem infarction	No MRI available MRI must target the correct segment(s) of the spine.	False normal CT in early stroke	EEG often not available in the emergency department. Not performing LP in seizure patients who may have encephalitis or neurocysticercosis.

Preconceived notions	Headache improved with triptans so is not a serious secondary cause.	Posterior circulation strokes are obvious or devastating events	All patients with SEA have risk factors or fever, or neurological deficits	Young people do not get strokes	Seizures (or seizure-like movements) are sometimes seen with strokes. Convulsive movements are common in syncope.

CVST: cerebral venous sinus thrombosis, SAH: subarachnoid hemorrhage, CT: CAT scan, MRI: magnetic resonance imaging, SEA: spinal epidural abscess.

## References

[1] Pitts SR, Niska RW, Xu J, Burt CW (2008). National Hospital Ambulatory Medical Care Survey: 2006 emergency department summary. *National Health Statistics Reports*.

[2] Hansen CK, Fisher J, Joyce N, Edlow JA (2011). Emergency department consultations for patients with neurological emergencies. *European Journal of Neurology*.

[3] Moeller JJ, Kurniawan J, Gubitz GJ, Ross JA, Bhan V (2008). Diagnostic accuracy of neurological problems in the emergency department. *Canadian Journal of Neurological Sciences*.

[4] Moulin T, Sablot D, Vidry E (2003). Impact of emergency room neurologists on patient management and outcome. *European Neurology*.

[5] Rizos T, Juttler E, Sykora M, Poli S, Ringleb PA (2011). Common disorders in the neurological emergency room—experience at a tertiary care hospital. *European Journal of Neurology*.

[6] Royl G, Ploner CJ, Möckel M, Leithner C (2010). Neurological chief complaints in an emergency room. *Nervenarzt*.

[7] Falco FA, Sterzi R, Toso V (2008). The neurologist in the emergency department. An Italian nationwide epidemiological survey. *Neurological Sciences*.

[8] Wu CL, Wang FT, Chiang YC (2010). Unplanned emergency department revisits within 72 hours to a secondary teaching referral hospital in Taiwan. *Journal of Emergency Medicine*.

[9] Goldstein JN, Camargo CA, Pelletier AJ, Edlow JA (2006). Headache in United States emergency departments: demographics, work-up and frequency of pathological diagnoses. *Cephalalgia*.

[10] Edlow JA, Panagos PD, Godwin SA, Thomas TL, Decker WW (2008). Clinical policy: critical issues in the evaluation and management of adult patients presenting to the emergency department with acute headache. *Annals of Emergency Medicine*.

[11] Edlow JA, Malek AM, Ogilvy CS (2008). Aneurysmal subarachnoid hemorrhage: update for emergency physicians. *Journal of Emergency Medicine*.

[12] Edlow JA, Caplan LR (2000). Avoiding pitfalls in the diagnosis of subarachnoid hemorrhage. *The New England Journal of Medicine*.

[13] Vermeulen MJ, Schull MJ (2007). Missed diagnosis of subarachnoid hemorrhage in the emergency department. *Stroke*.

[14] Bø SH, Davidsen EM, Gulbrandsen P, Dietrichs E (2008). Acute headache: a prospective diagnostic work-up of patients admitted to a general hospital. *European Journal of Neurology*.

[15] Pope JV, Edlow JA (2008). Favorable response to analgesics does not predict a benign etiology of headache. *Headache*.

[16] Evans RW, Johnston JC (2011). Migraine and medical malpractice. *Headache*.

[17] Perry JJ, Stiell IG, Sivilotti MLA (2011). Sensitivity of computed tomography performed within six hours of onset of headache for diagnosis of subarachnoid haemorrhage: prospective cohort study. *BMJ*.

[18] Kowalski RG, Claassen J, Kreiter KT (2004). Initial misdiagnosis and outcome after subarachnoid hemorrhage. *JAMA*.

[19] Jones NS (2009). Sinus headaches: avoiding over-and mis-diagnosis. *Expert Review of Neurotherapeutics*.

[20] Mudgil SP, Wise SW, Hopper KD, Kasales CJ, Mauger D, Fornadley JA (2002). Correlation between presumed sinusitis-induced pain and paranasal sinus computed tomographic findings. *Annals of Allergy, Asthma and Immunology*.

[21] Valentinis L, Tuniz F, Valent F (2010). Headache attributed to intracranial tumours: a prospective cohort study. *Cephalalgia*.

[22] Schankin CJ, Ferrari U, Reinisch VM, Birnbaum T, Goldbrunner R, Straube A (2007). Characteristics of brain tumour-associated headache. *Cephalalgia*.

[23] Shemie S, Jay V, Rutka J, Armstrong D (1997). Acute obstructive hydrocephalus and sudden death in children. *Annals of Emergency Medicine*.

[24] Bousser MG, Ferro JM (2007). Cerebral venous thrombosis: an update. *The Lancet Neurology*.

[25] Fusco MR, Harrigan MR (2011). Cerebrovascular dissections—a review part I: spontaneous dissections. *Neurosurgery*.

[26] Kerber KA (2009). Vertigo presentations in the emergency department. *Seminars in Neurology*.

[27] Newman-Toker DE (2007). Charted records of dizzy patients suggest emergency physicians emphasize symptom quality in diagnostic assessment. *Annals of Emergency Medicine*.

[28] Newman-Toker DE, Camargo CA, Hsieh YH, Pelletier AJ, Edlow JA (2009). Disconnect between charted vestibular diagnoses and emergency department management decisions: a cross-sectional analysis from a nationally representative sample. *Academic Emergency Medicine*.

[29] Kerber KA, Brown DL, Lisabeth LD, Smith MA, Morgenstern LB (2006). Stroke among patients with dizziness, vertigo, and imbalance in the emergency department: a population-based study. *Stroke*.

[30] Cheung CSK, Mak PSK, Manley KV (2010). Predictors of important neurological causes of dizziness among patients presenting to the emergency department. *Emergency Medicine Journal*.

[31] Lawson J, Johnson I, Bamiou DE, Newton JL (2005). Benign paroxysmal positional vertigo: clinical characteristics of dizzy patients referred to a Falls and Syncope Unit. *QJM*.

[32] Newman-Toker DE, Camargo CA (2006). ‘Cardiogenic vertigo’—true vertigo as the presenting manifestation of primary cardiac disease. *Nature Clinical Practice Neurology*.

[33] Kattah JC, Talkad AV, Wang DZ, Hsieh YH, Newman-Toker DE (2009). HINTS to diagnose stroke in the acute vestibular syndrome: three-step bedside oculomotor examination more sensitive than early MRI diffusion-weighted imaging. *Stroke*.

[34] Lee H, Sohn SI, Cho YW (2006). Cerebellar infarction presenting isolated vertigo: frequency and vascular topographical patterns. *Neurology*.

[35] Kerber KA, Morgenstern LB, Meurer WJ (2011). Nystagmus assessments documented by emergency physicians in acute dizziness presentations: a target for decision support?. *Academic Emergency Medicine*.

[36] Royl G, Ploner CJ, Leithner C (2011). Dizziness in the emergency room: diagnoses and misdiagnoses. *European Neurology*.

[37] Kim AS, Fullerton HJ, Johnston SC (2011). Risk of vascular events in emergency department patients discharged home with diagnosis of dizziness or vertigo. *Annals of Emergency Medicine*.

[38] Savitz SI, Caplan LR, Edlow JA (2007). Pitfalls in the diagnosis of cerebellar infarction. *Academic Emergency Medicine*.

[39] Edlow JA, Newman-Toker DE, Savitz SI (2008). Diagnosis and initial management of cerebellar infarction. *The Lancet Neurology*.

[40] Deyo RA, Weinstein JN (2001). Low back pain. *The New England Journal of Medicine*.

[41] Corwell BN (2010). The emergency department evaluation, management, and treatment of back pain. *Emergency Medicine Clinics of North America*.

[42] Kostuik JP (2004). Medicolegal consequences of cauda equina syndrome: an overview. *Neurosurg Focus*.

[43] Jalloh I, Minhas P (2007). Delays in the treatment of cauda equina syndrome due to its variable clinical features in patients presenting to the emergency department. *Emergency Medicine Journal*.

[44] Bell DA, Collie D, Statham PF (2007). Cauda equina syndrome—what is the correlation between clinical assessment and MRI scanning?. *British Journal of Neurosurgery*.

[45] Domen PM, Hofman PA, Van Santbrink H, Weber WEJ (2009). Predictive value of clinical characteristics in patients with suspected cauda equina syndrome. *European Journal of Neurology*.

[46] Dugas AF, Lucas JM, Edlow JA (2011). Diagnosis of spinal cord compression in nontrauma patients in the emergency department. *Academic Emergency Medicine*.

[47] Darouiche RO (2006). Spinal epidural abscess. *The New England Journal of Medicine*.

[48] Davis DP, Wold RM, Patel RJ (2004). The clinical presentation and impact of diagnostic delays on emergency department patients with spinal epidural abscess. *Journal of Emergency Medicine*.

[49] Pradilla G, Nagahama Y, Spivak AM, Bydon A, Rigamonti D (2010). Spinal epidural abscess: current diagnosis and management. *Current Infectious Disease Reports*.

[50] Reihsaus E, Waldbaur H, Seeling W (2000). Spinal epidural abscess: a meta-analysis of 915 patients. *Neurosurgical Review*.

[51] Tang HJ, Lin HJ, Liu YC, Li CM (2002). Spinal Epidural Abscess-Experience with 46 patients and evaluation of prognostic factors. *Journal of Infection*.

[52] Beronius M, Bergman B, Andersson R (2001). Vertebral osteomyelitis in Göteborg, Sweden: a retrospective study of patients during 1990–95. *Scandinavian Journal of Infectious Diseases*.

[53] Chelsom J, Solberg CO (1998). Vertebral osteomyelitis at a Norwegian university hospital 1987–97: clinical features, laboratory findings and outcome. *Scandinavian Journal of Infectious Diseases*.

[54] Deyo RA, Diehl AK (1988). Cancer as a cause of back pain: frequency, clinical presentation, and diagnostic strategies. *Journal of General Internal Medicine*.

[55] Lury K, Smith JK, Castillo M (2006). Imaging of Spinal Infections. *Seminars in Roentgenology*.

[56] El Sayed M, Witting MD (2011). Low yield of ED magnetic resonance imaging for suspected epidural abscess. *American Journal of Emergency Medicine*.

[57] McGillicuddy DC, Walker O, Shapiro NI, Edlow JA (2006). Guillain-Barré syndrome in the emergency department. *Annals of Emergency Medicine*.

[58] Winer JB (2008). Guillain-Barré syndrome. *BMJ*.

[59] van Doorn PA, Ruts L, Jacobs BC (2008). Clinical features, pathogenesis, and treatment of Guillain-Barré syndrome. *The Lancet Neurology*.

[60] Titulaer MJ, Lang B, Verschuuren JJGM (2011). Lambert-Eaton myasthenic syndrome: from clinical characteristics to therapeutic strategies. *The Lancet Neurology*.

[61] Scherer K, Bedlack RS, Simel DL (2005). Does this patient have myasthenia gravis?. *JAMA*.

[62] Smulowitz PB, Zeller J, Sanchez LD, Edlow JA (2005). Myasthenia gravis: lessons for the emergency physician. *European Journal of Emergency Medicine*.

[63] Frohman EM, Wingerchuk DM (2010). Transverse myelitis. *The New England Journal of Medicine*.

[64] Louis MES, Peck SHS, Bowering D (1988). Botulism from chopped garlic: delayed recognition of a major outbreak. *Annals of Internal Medicine*.

[65] Edlow JA (2010). Tick paralysis. *Current Treatment Options in Neurology*.

[66] Ferro JM, Pinto AN, Falcão I (1998). Diagnosis of Stroke by the Nonneurologist a validation study. *Stroke*.

[67] Kothari RU, Brott T, Broderick JP, Hamilton CA (1995). Emergency physicians: accuracy in the diagnosis of stroke. *Stroke*.

[68] Libman RB, Wirkowski E, Alvir J, Rao TH (1995). Conditions that mimic stroke in the emergency department: implications for acute stroke trials. *Archives of Neurology*.

[69] Morgenstern LB, Lisabeth LD, Mecozzi AC (2004). A population-based study of acute stroke and TIA diagnosis. *Neurology*.

[70] Newman-Toker DE, Robinson KA, Edlow JA (2008). Frontline misdiagnosis of cerebrovascular events in the era of modern neuroimaging: a systematic review. *Annals of Neurology*.

[71] Rizos T, Ringleb PA, Huttner HB, Köhrmann M, Jüttler E (2009). Evolution of stroke diagnosis in the emergency room—a prospective observational study. *Cerebrovascular Diseases*.

[72] Hand PJ, Kwan J, Lindley RI, Dennis MS, Wardlaw JM (2006). Distinguishing between stroke and mimic at the bedside: the brain attack study. *Stroke*.

[73] Goldstein LB, Simel DL (2005). Is this patient having a stroke?. *JAMA*.

[74] Nor AM, Davis J, Sen B (2005). The Recognition of Stroke in the Emergency Room (ROSIER) scale: development and validation of a stroke recognition instrument. *The Lancet Neurology*.

[75] Kuruvilla A, Bhattacharya P, Rajamani K, Chaturvedi S (2011). Factors associated with misdiagnosis of acute stroke in young adults. *Journal of Stroke and Cerebrovascular Diseases*.

[76] Nakajima M, Hirano T, Uchino M (2008). Patients with acute stroke admitted on the second visit. *Journal of Stroke and Cerebrovascular Diseases*.

[77] Martin-Schild S, Albright KC, Tanksley J (2011). Zero on the NIHSS does not equal the absence of stroke. *Annals of Emergency Medicine*.

[78] Edlow JA, Selim MH (2011). Atypical presentations of acute cerebrovascular syndromes. *The Lancet Neurology*.

[79] Johnston SC, Gress DR, Browner WS, Sidney S (2000). Short-term prognosis after emergency department diagnosis of TIA. *JAMA*.

[80] Prabhakaran S, Silver AJ, Warrior L, McClenathan B, Lee VH (2008). Misdiagnosis of transient ischemic attacks in the emergency room. *Cerebrovascular Diseases*.

[81] Schrock JW, Glasenapp M, Victor A, Losey T, Cydulka RK (2011). Variables associated with discordance between emergency physician and neurologist diagnoses of transient ischemic attacks in the emergency department. *Annals of Emergency Medicine*.

[82] Chowdhury FA, Nashef L, Elwes RDC (2008). Misdiagnosis in epilepsy: a review and recognition of diagnostic uncertainty. *European Journal of Neurology*.

[83] Josephson CB, Rahey S, Sadler RM (2007). Neurocardiogenic syncope: frequency and consequences of its misdiagnosis as epilepsy. *Canadian Journal of Neurological Sciences*.

[84] Francis P, Baker GA (1999). Non-epileptic attack disorder (NEAD): a comprehensive review. *Seizure*.

[85] Petkar S, Cooper P, Fitzpatrick AP (2006). How to avoid a misdiagnosis in patients presenting with transient loss of consciousness. *Postgraduate Medical Journal*.

[86] Carreño M (2008). Recognition of nonepileptic events. *Seminars in Neurology*.

[87] Kanner AM (2008). Common errors made in the diagnosis and treatment of epilepsy. *Seminars in Neurology*.

[88] Cooper PN, Westby M, Pitcher DW, Bullock I (2011). Synopsis of the national institute for health and clinical excellence guideline for management of transient loss of consciousness. *Annals of Internal Medicine*.

[89] Fitzpatrick AP, Cooper P (2006). Diagnosis and management of patients with blackouts. *Heart*.

[90] Oliva M, Pattison C, Carino J, Roten A, Matkovic Z, O’Brien TJ (2008). The diagnostic value of oral lacerations and incontinence during convulsive "seizures". *Epilepsia*.

[91] Sheldon R, Rose S, Ritchie D (2002). Historical criteria that distinguish syncope from seizures. *Journal of the American College of Cardiology*.

[92] Bakes KM, Faragher J, Markovchick VJ, Donahoe K, Haukoos JS (2011). The Denver Seizure Score: anion gap metabolic acidosis predicts generalized seizure. *American Journal of Emergency Medicine*.

[93] Stone J (2011). Functional neurological symptoms. *Journal of the Royal College of Physicians of Edinburgh*.

[94] Glick TH, Workman TP, Gaufberg SV (2000). Suspected conversion disorder: foreseeable risks and avoidable errors. *Academic Emergency Medicine*.

[95] McWhirter L, Stone J, Sandercock P, Whiteley W (2011). Hoover's sign for the diagnosis of functional weakness: a prospective unblinded cohort study in patients with suspected stroke. *Journal of Psychosomatic Research*.

[96] Chen Y, Bogosavljevic V, Leys D, Jovanovic D, Beslac-Bumbasirevic L, Lucas C (2011). Intravenous thrombolytic therapy in patients with stroke mimics: baseline characteristics and safety profile. *European Journal of Neurology*.

[97] Tsivgoulis G, Alexandrov AV, Chang J (2011). Safety and outcomes of intravenous thrombolysis in stroke mimics: a 6-year, single-care center study and a pooled analysis of reported series. *Stroke*.

[98] Artto V, Putaala J, Strbian D (2012). Stroke mimics and intravenous thrombolysis. *Annals of Emergency Medicine*.

[99] Stone J, Smyth R, Carson A (2005). Systematic review of misdiagnosis of conversion symptoms and ‘hysteria’. *British Medical Journal*.

[100] Brown TW, McCarthy ML, Kelen GD, Levy F (2010). An epidemiologic study of closed emergency department malpractice claims in a national database of physician malpractice insurers. *Academic Emergency Medicine*.

[101] Kachalia A, Gandhi TK, Puopolo AL (2007). Missed and delayed diagnoses in the emergency department: a study of closed malpractice claims from 4 liability insurers. *Annals of Emergency Medicine*.

[102] Newman-Toker DE, Pronovost PJ (2009). Diagnostic errors the next frontier for patient safety. *JAMA*.

[103] Kocher KE, Meurer WJ, Fazel R, Scott PA, Krumholz HM, Nallamothu BK (2011). National trends in use of computed tomography in the emergency department. *Annals of Emergency Medicine*.

[104] Prevedello LM, Raja AS, Zane RD (2012). Variation in use of head computed tomography by emergency physicians. *American Journal of Medicine*.

[105] Smith-Bindman R (2010). Is computed tomography safe?. *The New England Journal of Medicine*.

[106] Edlow JA (2010). What are the unintended consequences of changing the diagnostic paradigm for subarachnoid hemorrhage after brain computed tomography to computed tomographic angiography in place of lumbar puncture?. *Academic Emergency Medicine*.

[107] Kassirer JP (1989). Our stubborn quest for diagnostic certainty: a cause of excessive testing. *The New England Journal of Medicine*.

[108] Glick TH (2005). The neurologist and patient safety. *Neurologist*.

[109] Stettler BA, Jauch EC, Kissela B, Lindsell CJ (2005). Neurologic education in emergency medicine training programs. *Academic Emergency Medicine*.

[110] Croskerry P, Nimmo GR (2011). Better clinical decision making and reducing diagnostic error. *Journal of the Royal College of Physicians of Edinburgh*.

[111] Eva KW, Link CL, Lutfey KE, McKinlay JB (2010). Swapping horses midstream: factors related to physicians’ changing their minds about a diagnosis. *Academic Medicine*.

[112] Graber ML (2009). Educational strategies to reduce diagnostic error: can you teach this stuff?. *Advances in Health Sciences Education*.

[113] Kassirer JP (2010). Teaching clinical reasoning: case-based and coached. *Academic Medicine*.

[114] Trowbridge RL (2008). Twelve tips for teaching avoidance of diagnostic errors. *Medical Teacher*.

[115] Wears RL (2009). What makes diagnosis hard?. *Advances in Health Sciences Education*.

[116] Scott IA (2009). Errors in clinical reasoning: causes and remedial strategies. *BMJ*.

[117] Schattner A, Magazanik N, Haran M (2010). The hazards of diagnosis. *QJM*.

